# Crawling wounded: molecular genetic insights into wound healing from *Drosophila* larvae

**DOI:** 10.1387/ijdb.180085mg

**Published:** 2018

**Authors:** CHANG-RU TSAI, YAN WANG, MICHAEL J. GALKO

**Affiliations:** 1 Program in Developmental Biology, Baylor College of Medicine, University of Texas MD Anderson Cancer Center, Houston, Texas, USA; 2 Department of Genetics, University of Texas MD Anderson Cancer Center, University of Texas MD Anderson Cancer Center, Houston, Texas, USA; 3 Genetics and Epigenetics Graduate Program, University of Texas MD Anderson Cancer Center, Houston, Texas, USA

**Keywords:** wound healing, cell-cell fusion, actin, signaling pathway, Drosophila

## Abstract

For animals, injury is inevitable. Because of this, organisms possess efficient wound healing mechanisms that can repair damaged tissues. However, the molecular and genetic mechanisms by which epidermal repair is accomplished remain poorly defined. *Drosophila* has become a valuable model to study epidermal wound healing because of the comprehensive genetic toolkit available in this organism and the similarities of wound healing processes between *Drosophila* and vertebrates. Other reviews in this Special Issue cover wound healing assays and pathways in *Drosophila* embryos, pupae and adults, as well as regenerative processes that occur in tissues such as imaginal discs and the gut. In this review, we will focus on the molecular/genetic control of wound-induced cellular processes such as inflammation, cell migration and epithelial cell-cell fusion in *Drosophila* larvae. We will give a brief overview of the three wounding assays, pinch, puncture, and laser ablation, and the cellular responses that ensue following wounding. We will highlight the actin regulators, signaling pathways and transcriptional mediators found so far to be involved in larval epidermal wound closure and what is known about how they act. We will also discuss wound-induced epidermal cell-cell fusion and possible directions for future research in this exciting system.

## Cellular processes in different wounding assays in *Drosophila* larvae

*Drosophila* has a long history of fundamental discovery related to developmental biology (Nüsslein-Volhard and Wieschaus, 1980; Wangler *et al.*, 2015). So it was not surprising that the first studies to look at tissue repair in *Drosophila* exploited a developmentally-programmed morphogenetic event, dorsal closure (DC). During DC, two apposed epidermal sheets migrate dorsally and close a dorsal gap to form a continuous epidermal layer around the developing embryo (Harden, 2002; Young *et al.*, 1993). DC, because of its amenability to videomicroscopy, has become a classical model to study epithelial sheet migration and the physical forces that drive morphogenesis (Hayes and Solon, 2017). One issue that arose with this model, however, is that it does not involve any cellular responses to tissue damage-a hallmark of actual physiological wound healing. Thus, other models that possessed this feature were developed in embryos, larvae, pupae, and adults.

The larval epidermis is a monolayer of post-mitotic epithelial cells. These cells continue to endoreplicate their DNA content through out the larval stages (Smith and Orr-Weaver, 1991; Wang *et al.*, 2015) and grow to a substantial size (up to 50 μm across). A major function of these cells is to manufacture and secrete the cuticle that forms the larval exoskeleton and permeability barrier. Apically, larval epidermal cells possess microvilli believed to be specialized for cuticle assembly/secretion (Gangishetti *etal.*, 2012; Payre, 2004) and basally they possess a basal lamina (Fessler *et al.*, 1994) that separates them from the hemolymph filling the larval body cavity. Floating within this open circulatory system is a population of circulating innate immune hemocytes (blood cells) (Rizki and Rizki, 1980) that are potentially damage-responsive.

**Fig. 1.**
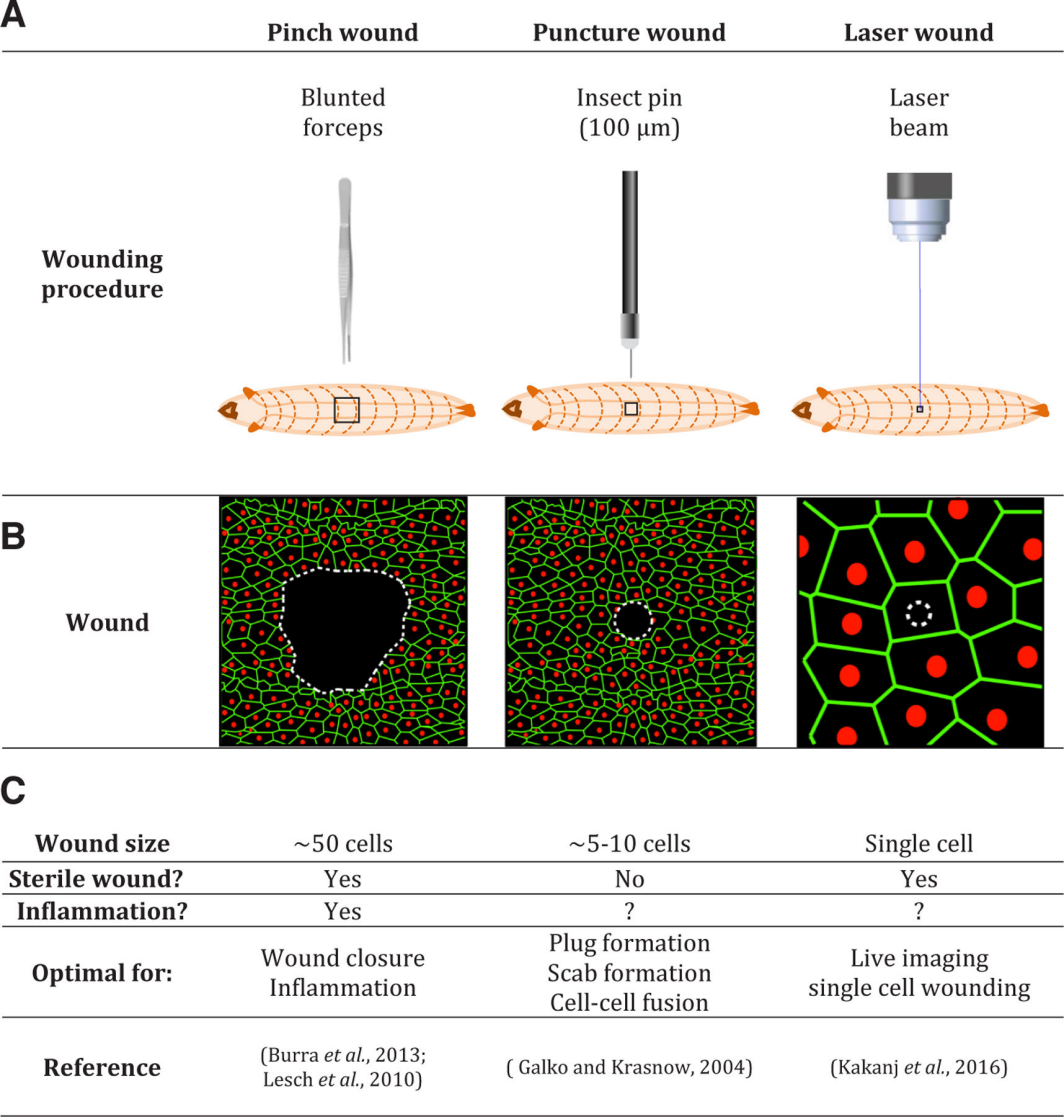
Methods used to wound *Drosophila* larvae. **(A)** Cartoons of the different wounding procedures. **(B)** Schematics of the larval epithelia after wounding. Membranes, green; Nuclei, red. White dashed lines indicate the wound edge. **(C)** Details of size, sterility, inflammation, optimal uses, and primary references.

A number of assays have been developed to examine cellular responses to damage in larvae. (Fig. 1) (Burra *et al.*, 2013; Galko and Krasnow, 2004; Kakanj *et al.*, 2016). Puncture wounding uses a 100 *μ*m diameter needle to pierce through both the cuticle barrier and the underlying epidermis (Galko and Krasnow, 2004). Punctured larvae bleed and form a melanized plug that prevents blood (hemolymph) loss. The process of sealing the wound site likely combines melanization (Bidla *et al.*, 2005) with a protein-based hemolymph clotting system (Scherfer *et al.*, 2004). Successful plug formation and survival after puncture wounding is dependent upon factors provided by a specialized set of hemocytes, crystal cells (Bidla *et al.*, 2007; Meister, 2004; Rizki *et al.*, 1985). Crystal cells, named after cystoplasmic crystalline deposits, promote the scab formation at wounds. These wounds also induce formation of a specialized blood cell type, lamellocytes (Márkus *et al.*, 2005) that are typically involved in responses to parasitic wasps (Rizki and Rizki, 1992). The epidermal cells surrounding the plug orient towards it and in many cases fuse to form a syncytium (cell-cell fusion will be discussed further below). As these cell rearrangements are occurring, wound-edge epidermal cells extend long lamellar cellular processes and migrate across the wound gap to reestablish a continuous polarized epithelium. The puncture wound procedure is an excellent assay to study plug and scab formation as well as cell-cell fusion and other cell shape changes.

A second wounding procedure creates scabless wounds. In this method blunted forceps are used to gently pinch larvae on the dorsal aspect of an abdominal segment (Burra *et al.*, 2013). This procedure leaves the overlying cuticle intact but interrupts the continuity of the underlying epidermal sheet. Because the cuticle is not disrupted, pinched larvae do not bleed and do not form a plug and scab at the wound site. These wounds are thus sterile and allow the investigator to probe tissue damage-associated responses in the absence of cross-talk from infection. As with the smaller puncture wounds, wound-edge epidermal cells orient towards the wound, form syncytia (Galko and Krasnow, 2004), and elongate into the wound gap (Lesch *et al.*, 2010; Wu *et al.*, 2009). Within 24 hours, all the pinch-wounded animals have closed their wounds. Pinch wounding abrades a larger patch of the epidermal cells compared to the puncture technique. Because the wound site can be clearly viewed through the transparent and unmelanized cuticle, this procedure is a powerful model to study the genetic basis of wound closure (Lesch *et al.*, 2010). However, the long timecourse of healing and the large area over which it occurs makes live-imaging of these wounds a substantial challenge. Interestingly, the shapes of individual cells change dramatically during wound healing. Before wounding cells are mostly pentagonal or hexagonal. However, more and more cells become irregularly shaped as the wounds are healed (Kwon *et al.*, 2010).

Epidermal cells are not the only cells that are wound-responsive in larvae following pinch wounding. In addition, larvae possess macrophage-like cells called plasmatocytes that circulate within the open body cavity. Within a few hours after pinch wounding, those circulating plasmatocytes that encounter the wound attach to it. These cells then change their shape from approximately spherical to a spread morphology and contribute to phagocytosis of the cell debris at the wound sites (Babcock *et al.*, 2008). The mechanisms of plasmatocyte recruitment to embryonic (Stramer *et al.*, 2005) and pupal (Moreira *et al.*, 2011) wounds are quite different, involving predominantly cell migration responses (Brock *et al.*, 2008). In larvae, inflammation, as assessed by plasmatocyte recruitment, has only been examined in detail at pinch wounds.

A newcomer to the suite of wounding procedures used in larvae is laser-induced tissue damage (Kakanj *et al.*, 2016). Lasers have long been used in the embryo (Wood *et al.*, 2002). In larvae, lasers make wounds at essentially at the single cell level. These wounds activate responses in viable cells that surround the ablated cell. They heal more quickly than larger pinch and puncture wounds and thus permit live visualization of the spatially-restricted healing response. This is an advance because live imaging of puncture-or pinch-wounded larvae has been challenging due to local melanization (puncture wounds) and the difficulty of immobilizing larvae for over the full course of healing (pinch wounds).

## Actin regulators that execute different cell behaviors during wound healing

Because pinch wounding creates a larger wound gap than the other wounding techniques, more epidermal cells around the wound participate in the healing process. A hallmark of wound healing in *Drosophila* larvae is that cell proliferation and apoptosis do not play essential roles during this process (Lesch *et al.*, 2010; Tsai *et al.*, 2017; Wang *et al.*, 2015). This differs from wound healing responses in most vertebrate epithelia (Park *et al.*, 2017; Wang *et al.*, 2017) but offers the opportunity to study wound closure that is not driven in part by proliferative generation of new cells. In larvae, epidermal cells cover the wound gap by the combined actions of cell migration and cell growth. A result of these cellular responses is that the engaged epidermal cells change dramatically in shape during the first several hours of healing (Galko and Krasnow, 2004; Kwon *et al.*, 2010). Within a few hours after wounding the leading-edge cells send out cellular processes toward the wound center (Wu *et al.*, 2009), whereas the cells behind these follow and change their shapes as they do so (Lesch *et al.*, 2010). The cellular behaviors of leading-edge cells and followers are similar to the collective migration observed in cell culture (Lesch *et al.*, 2010; Simpson *et al.*, 2008; Vitorino and Meyer, 2008).

**Fig. 2.**
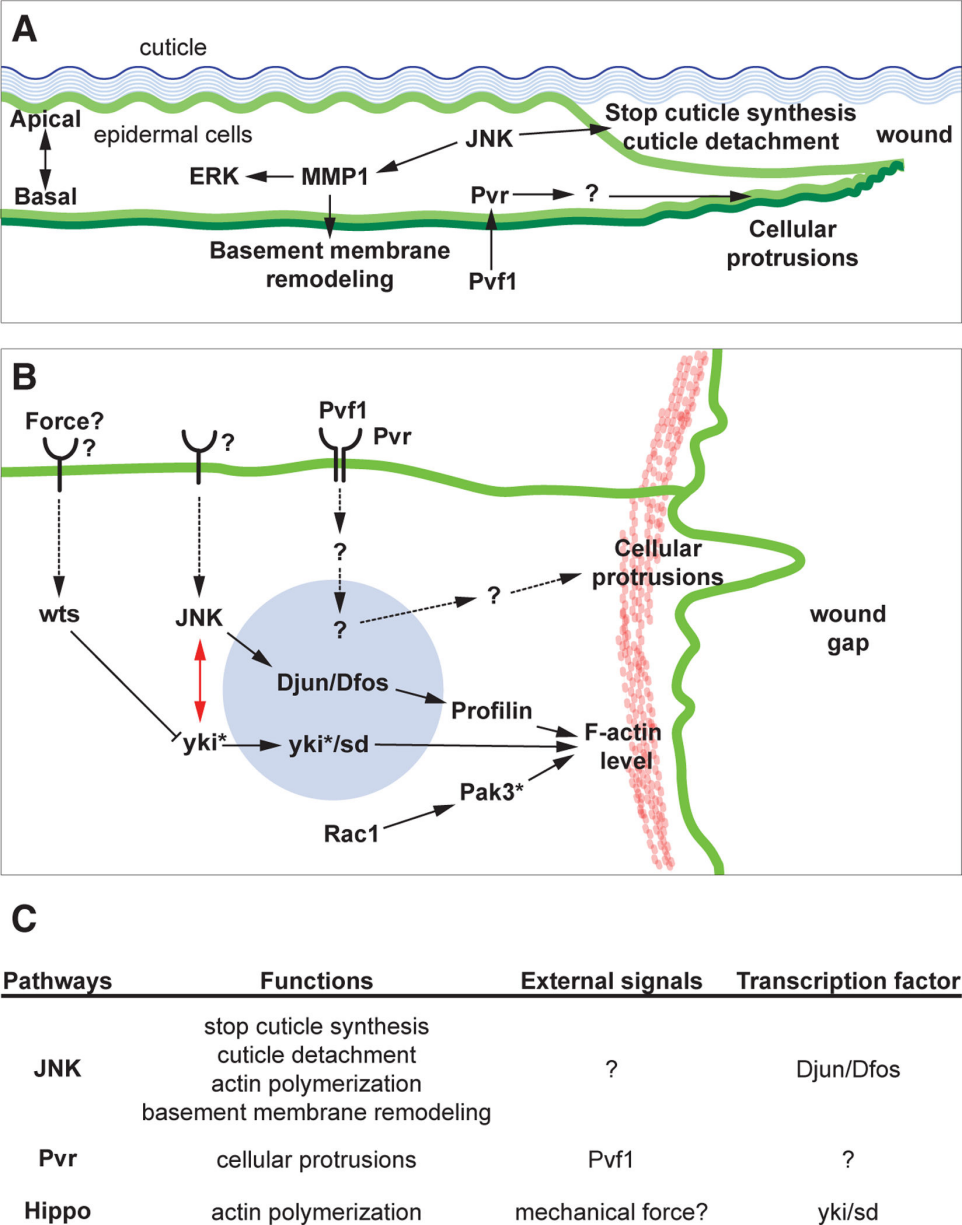
Signaling pathways that regulate cellular responses in wound-edge cells. **(A)** Cartoon of transverse view of a wound edge larval epidermal cell annotated with cellular structures and functions of wound healing pathways. **(B)** Cartoon of top-down view of a wound edge larval epidermal cell. Receptors and ligands that affect pinch wounding are illustrated as are selected pathway components, in particular transcription factors and target genes that regulate actin. Double-headed red arrow indicates the genetic interaction between JNK and yki signaling. Other pathway interactions or lack thereof are addressed in the text. Asterisk: proteins that show translocation after wounding. **(C)** Table summarizing wound healing pathway functions, signals, and transcription factors, if known. The role of insulin signaling in healing of multicellular wounds is not yet clear and is not depicted here-please refer to the section on insulin signaling for its roles in single cell healing in the larval epidermis.

Cell shape changes and cell migration require cytoskeletal remodeling (Brüser and Bogdan, 2017). Consistent with this, several actin regulators have been found to play critical roles during wound healing (Table 1). Epidermal cells around the wound margin detach from the cuticle nearest to the wound edge and send out long cell protrusions (both filopodia and lamellipodia) within a few hours of pinch wounding (Wu *et al.*, 2009) (Fig. 2A). Indeed, levels of filamentous actin rapidly increase around the wound margin (Kakanj *et al.*, 2016; Kwon *et al.*, 2010; Wu *et al.*, 2009) and several actin regulators are required for this increased actin (Baek *et al.*, 2010; Baek *et al.*, 2012; Brock *et al.*, 2012; Kakanj *et al.*, 2016) (Fig. 2B). The actin cable observed a few hours after pinch wounding is discontinuous (Brock *et al.*, 2012), whereas a continuous actomyosin cable that surrounds the wounds was observed in the laser-wounded epidermis (Kakanj *et al.*, 2016).

A cytoskeleton regulator that acts downstream of Rac1, P21-activated kinase 3 (Pak3), translocates to the wound margin a few hours after wounding and is required for both wound closure and up-regulation of filamentous actin around the wound margin (Baek *et al.*, 2012). Similarly, a regulator of actin turnover dynamics– Profilin, encoded by *chickadee* (*chic*) in *Drosophila*, is also required for epidermal wound closure. Profilin protein levels increase surrounding the wound throughout and following active closure. Loss of *chic* reduces actin polymerization around the wound (Brock *et al.*, 2012). Interestingly, RNAi transgenes targeting *Drosophila yorkie (yki), a* YAP-like transcriptional activator, and *scalloped (sd)*, a TEA Domain family protein-like transcription factor (TEAD), common transcriptional mediators of the Hippo pathway, also cause reduced actin polymerization around the wound edge and wound closure defects (Tsai *et al.*, 2017).

Similar to DC, the *Drosophila* non-muscle myosin II heavy chain, encoded by *zipper (zip)*, is also required for wound closure (Kwon *et al.*, 2010). Larvae with the myosin II heavy chain knocked down by RNAi in the epidermis show altered cell shape distributions. Normally cells around the wound assume irregular shapes whereas those lacking *zipper* stay relatively polygonal. Furthermore, both the myosin II heavy chain and the light chain, encoded by *spaghetti squash*, translocate to the rear end of the cell cortex in the first few rows of epidermal cells around the wound edge, leading to a polarized non-muscle myosin II subcellular localization a few hours after wounding (Kwon *et al.*, 2010). Multiple Rho GTPases (Rac1, Cdc42 and Rho1) are required for Myosin II polarization (Baek *et al.*, 2010). As Rac1 and Cdc42 are required for wound closure (Lesch *et al.*, 2010), this may be an important signal to direct epidermal cell migration toward the center of the wound. Other known regulators of actin dynamics (*Arp10*, *Arp2*, *Gγ1*, *SCAR, mbc, Ced-12*) exhibit wound closure defects when targeted by RNAi in the larval epidermis (Lesch *et al.*, 2010) but their specific effects on actin or myosin localization have yet to be determined.

**Fig. 3.**
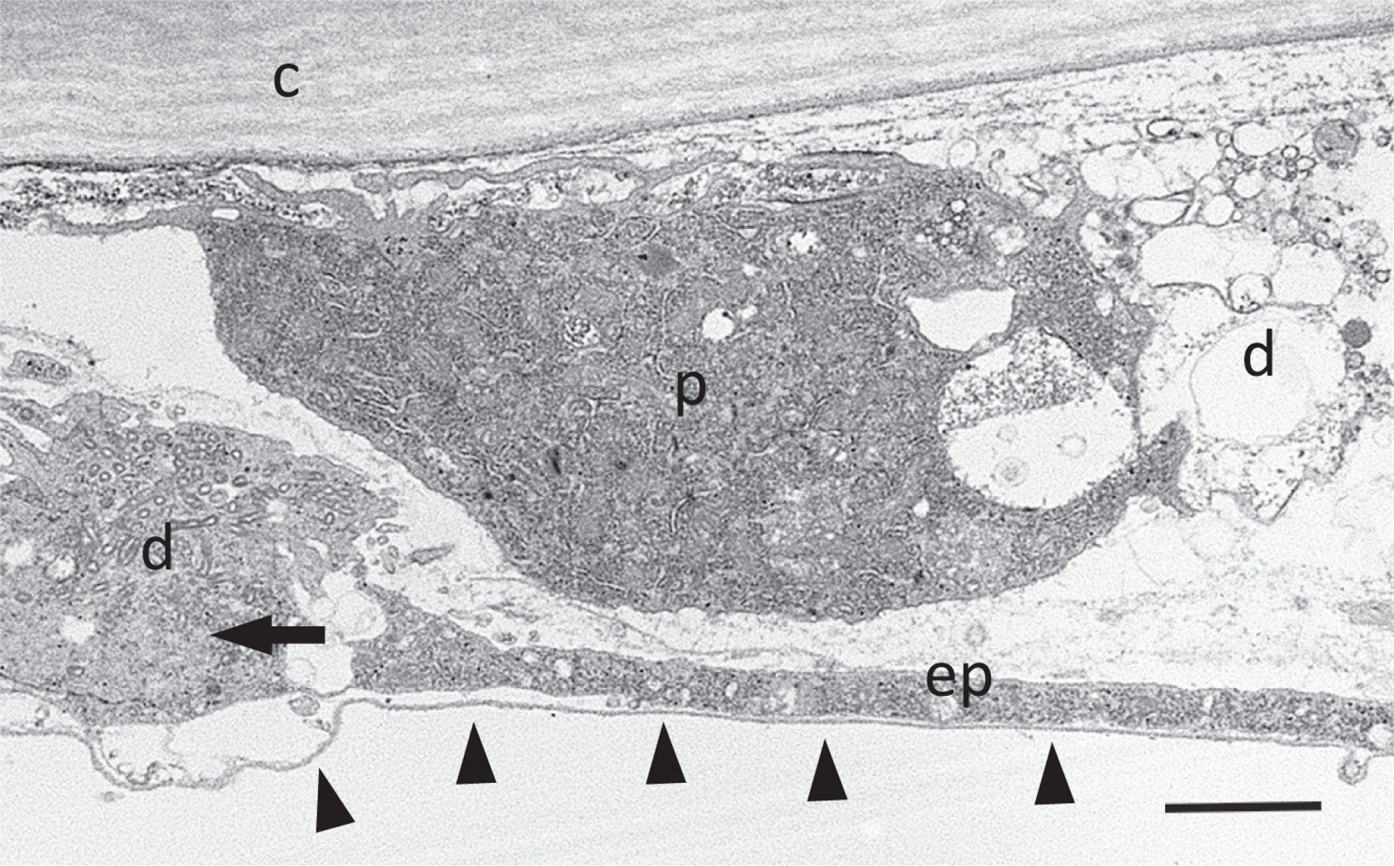
Wound-edge epidermal cell processes possess a basal lamina along their entire length. A transverse section of a whole-mount epidermis eight hours after wounding under transmission electron microscopy. The basal side of the thin process contains a basal lamina (arrowheads) to the end of the extension (arrow). ep, epidermal process; c, cuticle; d, wound site debris; p, plasmatocyte. Scale bar, 2 μm.

Basement membrane dynamics also appear to be important for proper closure. The long cellular processes that extend into the wound gap possess a basal lamina along their entire length even when they are very thin and even where the cell is detached from cuticle at the apical surface (Fig. 3). How this extension/stretching of the basal lamina is achieved is not clear. What is clear is that loss of the proteases matrix metalloprotease 1 and 2 (*MMP1* and *MMP2*) blocks puncture wound closure (Stevens and Page-McCaw, 2012). Moreover, *MMP1*, is required in the epidermis to promote reepithelialization by remodeling the basement membrane, facilitating cell elongation and actin cytoskeletal reorganization (Stevens and Page-McCaw, 2012). MMP1 induction around the wound requires Jun N-terminal kinase (JNK) signaling as does Profilin induction (Brock *et al.*, 2012). Below we discuss the various signaling pathways required for wound closure and what is known about their transcriptional targets.

## Signaling pathways that regulate wound healing

Multiple signaling pathways play important roles during wound healing in mammals (Eming *et al.*, 2014). Until recently (Pineda *et al.*, 2015), it has been difficult to visualize wound closure in mice and determine more precise phenotypes of such pathways. Analysis of orthologous pathways in *Drosophila*, in addition to basic gene discovery, is a place where fly wound healing studies can have a major impact. Indeed, several signaling pathways that are required for wound healing in larvae have clear orthologs and/or conserved functions in vertebrates. Here we summarize the diverse signaling pathways that act during larval wound closure (see also Table 1) and synthesize how they regulate different cellular functions at the wound (Fig. 2).

### Jun N-terminal Kinase (JNK) signaling

JNK is a member of the mitogen activated protein kinase (MAPK) family, and plays important roles during development, physiology and disease (Weston and Davis, 2007). JNK (encoded by *basket* in flies) was originally implicated in DC (Riesgo-Escovar *et al.*, 1996; Sluss *et al.*, 1996). *basket* mutant embryos fail to complete DC resulting in a phenotype where the embryo resembles an open basket. Similarly, JNK signaling is also required for thorax closure during metamorphosis (Zeitlinger and Bohmann, 1999). Interestingly, JNK signaling is rapidly activated around wound sites, as indicated by two pathway reporters, *msn-lacZ* and *puc-lacZ* (Galko and Krasnow, 2004*)*. The activation of the pathway suggested that it might be functionally required for closure and, indeed, epidermal expression of RNAi transgenes targeting several JNK pathway components, including JNK kinase kinase kinase (*Jun4K*, *misshapen*), JNK kinase kinase (*Jun3K*, *slipper*), JNK kinase (*Jun2K*, *hemipterous)*, *JNK* (*basket*), *DJun* (*Jun-related antigen*) and *DFos* (*kayak*), all led to a substantial impairment of wound closure (Galko and Krasnow, 2004; Lesch *et al.*, 2010).

What are the essential functions of JNK signaling during wound healing? RNAi transgenes targeting *JNK* caused a strong wound closure defect but did not abolish actin polymerization and cell protrusion around the wound (Galko and Krasnow, 2004; Wu *et al.*, 2009). However, expression of a dominant negative form of the JNK, which leads to a more potent block of function (Lesch *et al.*, 2010), did reduce actin polymerization around the wound margin (Kwon *et al.*, 2010). This latter study also showed that loss of JNK blocked polarization of non-muscle myosin II and epidermal cell shape changes following wounding. Knockdown of *JNK* also abolished wound-induced Mmp1 up-regulation, which is important for basement membrane remodeling and cell elongation (Stevens and Page-McCaw, 2012). Finally, JNK signaling is also important for leading-edge epidermal cells to either disassociate from the apical cuticle or to stop cuticle synthesis-events that appear to facilitate effective epidermal cell migration (Wu *et al.*, 2009). The combined effect of these diverse processes-on actin dynamics, basement membrane dynamics, cuticle adhesion, and gene expression (see Fig. 2 A–C and below) lead to a highly penetrant wound closure defect when JNK signaling is blocked. One important outstanding question is what genes act upstream of JNK activation within leading edge cells. The Rac1, Cdc42 and Rho1 GTPases can all activate JNK signaling in the unwounded epidermis although the strongest block of wound-induced JNK induction is observed with inhibition of Rac1 (Baek *et al*., 2010). A remaining question in both wound closure and DC is what is the external signal(s) that activate JNK signaling in these contexts.

### Platelet-derived growth factor and vascular endothelial growth factor-receptor related (Pvr) signaling

In mice, vascular Endothelial Growth Factor signaling primarily regulates angiogenesis during wound healing (Bao *et al.*, 2009). Knockout of *VEGF-A*, a pathway ligand, specifically in keratinocytes reduced angiogenesis and delayed wound healing (Rossiter, 2004). Interestingly, the *Drosophila* VEGFR homolog, Pvr (Cho *et al.*, 2002; Heino *et al.*, 2001) and one of its ligands, Pvf1, are required for epidermal wound closure (Wu *et al.*, 2009). In thorax closure during metamorphosis, Pvr signaling acts upstream of JNK signaling (Ishimaru *et al.*, 2004). However, in wound closure Pvr appears to act in parallel to JNK signaling (Fig. 2 A,B) because JNK reporters are activated at normal levels in the Pvr-deficient epidermis (Wu *et al.*, 2009). Interestingly, both Pvr/VEGFR and Pvf1/VEGF are functionally required in the epidermis, indicating that Pvf1/Pvr signaling acts in an autocrine fashion. The current model is that tissue damage/wounding exposes Pvr/VEGFR to ligand that is sequestered from the receptor in the unwounded state. This exposure then initiates epidermal cell migration (Wu *et al.*, 2009). Morphological comparison of wound-edge cells lacking Pvr/VEGFR indicated that Pvr/VEGFR is critical for cells to extend a cellular process into a wound gap. So far, the exact downstream mediators of Pvr/VEGFR signaling are yet to be identified. One common downstream factor of receptor tyrosine kinase (RTK) signaling, ERK/MAPK, was phosphorylated upon wounding (Wu *et al.*, 2009). However, this activation is not Pvr-dependent suggesting that the transduction pathway downstream of Pvr/ VEGFR may be non-canonical in some respects. Interestingly, Erk activation following wounding is MMP1-dependent (Stevens and Page-McCaw, 2012).

### Insulin and TOR signaling

Diabetic patients have reduced or abnormal insulin and TOR signaling, and often exhibit impaired wound healing (Eming *et al.*, 2014). Angiogenesis defects have been linked to compromised wound healing in diabetes (Okonkwo and Dipietro, 2017). However, it is not completely understood whether TOR and insulin signaling are required within the epidermis for normal wound closure. Using laser wounding in larvae (Fig. 1), the requirements of these signaling pathways were tested. Interestingly, insulin/FOXO and TOR/S6k signaling regulate epidermal wound healing in parallel, where insulin signaling activates actomyosin ring assembly but not glycogen metabolism (Kakanj *et al.*, 2016). Consistent with these findings, mammalian keratinocytes reduce their migratory capacity under high sugar conditions in culture (Lan *et al.*, 2008; Morita *et al.*, 2005; Song *et al.*, 2008). This suggests that a function of insulin and TOR signaling pathways during skin wound healing may be conserved between invertebrates and vertebrates, an idea supported by keratinocyte-specific deletion of FOXO1 in mice (Zhang *et al.*, 2015). It will be interesting to test if these signaling pathways are also required for healing pinch wounds since the main driving force for this type of wounding is cell migration rather than actomyosin-based contraction. It will also be interesting to determine whether insulin signaling is required for wound healing in the embryo, pupa, or adult. Potential interactions between insulin signaling and other wound healing pathways (JNK, Pvr, Yorkie) have yet to be examined.

### Hippo pathway

*Drosophila* YAP, which is encoded by *yorkie (yki)*, the transcriptional activator of the Hippo pathway, controls organ size and tissue regeneration through its well-characterized roles in balancing apoptosis (Huang *et al.*, 2005; Udan *et al.*, 2003) and cell division (Lin and Pearson, 2014). Interestingly, Yki and its binding partner, Scalloped (Sd– TEAD in mammals), are required for epidermal wound closure but they do this without balancing apoptosis and cell division in this tissue (Tsai *et al.*, 2017). Another recent study showed that Yki regulates cell shape in the tracheal system without effects on proliferation/apoptosis (Robbins *et al.*, 2014). Interestingly, Yki and Sd regulate actin polymerization at the wound edge to promote wound healing (Tsai *et al.*, 2017). In this context, overexpression of Warts (LATS in human) and Expanded (FRMD1 in human), two negative regulators of Yki, also blocked wound closure, indicating that at least part of the canonical Hippo pathway signaling cascade is involved in this process. Moreover, genetic analysis suggests that Yki interacts with the JNK pathway (See Fig. 2B) and likely acts downstream of or parallel to JNK signaling during wound closure (Tsai *et al.*, 2017).

During wound closure multiple cell behaviors such as cell migration and actin remodeling are activated. The signaling pathways discussed above control these responses in part through direct signaling effects but also likely through regulation of gene transcription and chromatin remodeling. Below we will discuss what is known about the regulation of gene expression during larval wound healing and the connection between transcriptional responses and signaling pathways required for closure. Other reviews in this Special Issue cover transcriptional events that accompany regeneration of other tissues such as imaginal discs and the adult gut epithelium.

## Transcriptional and epigenetic regulation during larval wound healing

Cuticle secretion to create a robust barrier is the main physiological function of larval epidermal cells. However, upon wounding the migrating front of leading edge epidermal cells transiently stops synthesizing cuticle (Wu *et al.*, 2009). This drastic cellular response and others such as cell shape changes are likely to be regulated at both the transcriptional and epigenetic levels. To determine whether epigenetic modifiers change expression levels upon wounding, fluorescently tagged reporters for different epigenetic factors were examined (Anderson and Galko, 2014). Seven regulators showed strongly diminished expression at the wound edges after wounding. Three down-regulated proteins– Osa, Kismet and Spt6, are generally associated with active chromatin (Andrulis *et al.*, 2000; Kaplan *et al.*, 2000; Kennison and Tamkun, 1988; Srinivasan, 2005; Srinivasan *et al.*, 2008), while four others, Sin3A, Sap130, Mi-2 and Mip120, are more associated with repressed chromatin (Ahringer, 2000; Ayer, 1999; Bernstein *et al.*, 2000; Fazzio *et al.*, 2001; Kehle *et al.*, 1998; Korenjak *et al.*, 2004; Lewis *et al.*, 2004; Spain *et al.*, 2010). The fast clearance of both positive and negative chromatin modifiers may allow wound-edge epidermal cells to alter their transcriptional response in a fairly global way after wounding (Anderson and Galko, 2014). Pvr and JNK signaling are not required for the clearances (Anderson and Galko, 2014), suggesting that other early wound signals exist. It will be interesting to test if other wound healing pathways are required for this clearance.

In addition to epigenetic regulators, several transcription factors are activated upon wounding and are necessary for wound closure (table 1). For example, DJun and DFos, the downstream transcription factors of the JNK signaling pathway, are also required for wound closure (Lesch *et al.*, 2010). This suggests that transcriptional responses are required for wound closure to proceed normally. Indeed, DFos is required for induction of a transcriptional target, Jun4K (*misshapen/*msn in flies), which is also important for normal wound closure (Lesch *et al.*, 2010). Moreover, DJun and DFos are both required to activate increased expression of the actin regulator, *Profilin*/*chic*, to promote wound healing (Brock *et al*., 2012). Finally, wound-induced *Mmp1* up-regulation is also JNK dependent (Stevens and Page-McCaw, 2012), though specific roles for DJun and DFos were not examined in this study. Presumably there are the other transcriptional targets of JNK signaling that are important for various aspects of wound closure and identifying these targets will be important moving forward.

Activation of the insulin receptor following wounding leads to the translocation of the transcription factor, Foxo, from the nucleus to the cytoplasm. This ensures proper actomyosin cable assembly, indicating that a normal function of Foxo is to suppress actomyosin cable assembly through as yet undefined targets and mechanisms (Kakanj *et al.*, 2016). In addition, Yki translocates to the nucleus in some of the epidermal cells around the wound (Tsai *et al.*, 2017). Both *yki* and *sd* are required for wound closure. However, the transcriptional targets of Yki/Sd that regulate actin cytoskeleton are still unknown in this context. Interestingly, transcription levels of several actin-related genes were up-regulated when *Salvador* (*Salv1*), which encodes a critical adaptor protein for Hippo activation, was conditionally knockout during heart regeneration in mice (Morikawa *et al.*, 2015). Also, YAP/TEAD activates of several migration-related genes in human cancer cell lines (Liu *et al.*, 2016). It will be interesting to know whether *yki/ sd* also activates these genes during larval wound closure. Finally, reporters that reflect the activity of Signal Transducer and Activator of Transcription (STAT) transcription factor are activated slightly later and further away from the wound center than JNK signaling (Lee *et al.*, 2017). Interestingly, loss-of-STAT reduced wound-induced integrin transcription and restricted cell-cell fusion in the vicinity of the healing wounds (Lee *et al.*, 2017). The next section discusses wound-induced cell-cell fusion in more detail.

While several transcription factors have been placed downstream of different signaling pathways, roles for other transcription factors (for instance is there a factor that acts downstream of Pvr signaling?) and the full suite of downstream functional targets remains to be identified. In addition, how the epigenetic changes observed upon wounding couple to wound-induced transcriptional regulation will be an intriguing topic to pursue.

## Wound-induced and genetic-induced epidermal cell-cell fusion

Multinucleate cells are observed near wounds within hours of wounding in larvae (Galko and Krasnow, 2004), pupae (Wang *et al.*, 2015) and adults (Losick *et al.*, 2013), suggesting that cell-cell fusion (syncytium formation) is a common cellular process during wound healing at most developmental stages in *Drosophila*. Although the function of cell-cell fusion during wound healing in larval and pupa is still unclear, cell-cell fusion appears to be critical for epidermal wound healing in adult flies (Losick *et al.*, 2013).

JNK signaling is activated with similar kinetics as the start of cell-cell fusion. Nevertheless, JNK signaling is not required for wound-induced cell-cell fusion (Galko and Krasnow, 2004; Wang *et al.*, 2015). To date, the signaling pathways that are required for wound-induced cell-cell fusion are still unknown. However, an important adhesion complex that suppressed cell-cell fusion has been reported, as has an interesting crosstalk with wound-induced JNK signaling. The integrin focal adhesion (FA) complex is critical for cells to bind to extracellular matrix as well as to send and receive signals (Legate *et al.*, 2009). Loss of *Integrin* β4α6 in mice leads to cell adhesion defects, epidermolysis bullosa and, neonatal death (Dowling *et al.*, 1996; Georges-Labouesse *et al.*, 1996). In *Drosophila* larvae, members of this complex also suppresses epithelial syncytium formation. Loss of the FA adaptor PINCH (particularly interesting new cysteine-histidine-rich protein), integrin-linked kinase (ILK), or β-integrin itself in the larval epidermis resulted in multinucleate epidermal cells even without wounding (Wang *et al.*, 2015). Interestingly, genetic reduction of integrin FA complex components, similar to wounding itself, also activated JNK signaling. This would appear to constitute a positive feedback loop as JNK signaling hyperactivation also disassembled integrin FA complex in larval epidermal cells (Wang *et al.*, 2015). Disassembly of the integrin FA complex was examined in both the wounded epidermis and upon JNK hyperactivation in the absence of wounding. In both cases, PINCH translocated from the plasma membrane to the cytoplasm, while ILK translocated from the plasma membrane to the nucleus (Wang *et al*., 2015). Presumably, the relocalized proteins cannot participate in functional adhesion. How FA complex disassembly is mediated at the mechanistic level and why disassembly should result in cell-cell fusion are open questions.

Epidermal cell-cell fusion seems to also involve transcriptional responses. Integrin levels increased dramatically after JNK signaling hyperactivation (Wang *et al*., 2015) or physical wounding (Lee *et al*., 2017). The actual mechanisms and functions of the integrin up-regulation are still not clear but increased Integrin expression may help stabilize the epidermis upon wounding or hyperactiva-tion of JNK signaling. Interestingly, loss of *Drosophila Jun4K* (*msn* in flies), which encodes an upstream kinase of the JNK signaling cascade, also increased wound-induced cell fusion (Lesch *et al.*, 2010). This suggests that Msn suppresses wound-induced cell-cell fusion, a different function from that observed upon JNK hyperactivation (Wang *et al.*, 2015).

Cell-cell fusion is also regulated by Janus kinase/Signal Transducer and Activator of Transcription (JAK/STAT) signaling (Lee *et al.*, 2017). JAK/STAT signaling is activated later than JNK signaling during wound healing. Like loss of integrin-FA components, loss of JAK/STAT signaling increased syncytial cell formation around pinch wounds, indicating a suppressive role in cell-cell fusion. This may provide a mechanism to spatially restrict cell-cell fusion as JNK activation and JAK-STAT activation occur in different spatial domains. JNK signaling is activated in the first few rows of cells bordering the wound, whereas JAK/STAT signaling is activated more distally (Lee *et al.*, 2017). Indeed, wound-induced cell-cell fusion primarily occurs in the JNK activation domain rather than JAK/STAT activation domain. Interestingly, this study also found that integrin is one of the downstream targets of JAK/STAT signaling upon wounding (Lee *et al.*, 2017). However, wound-induced integrin expression was detected within the JNK activation domain prior to the activation of JAK/STAT signaling. Therefore, it is likely that control of wound-induced integrin expression requires more inputs than solely JAK/STAT signaling.

## Future directions - sensing a wound and initiation of wound healing

Although certain genes and signaling pathways are crucial for larval wound healing, many open questions remain. One of the fundamental questions, for any repair process, ishow does the tissue first sense the wound and initiate appropriate wound responses? JNK signaling is rapidly turned on after wounding (Galko and Kras-now, 2004) and loss-of-*JNK* affects cuticle detachment, migration efficiency, and abolishes cell shape changes in both leading edge and follower cells (Kwon *et al.*, 2010; Lesch *et al.*, 2010). Thus, JNK signaling is a decent candidate for an early signal produced upon wounding. The main question here, and one that still persists even with respect to DC, is what external signal activates the JNK cascade? Attractive candidates include soluble signals known to activate JNK signaling in other contexts (Igaki *et al.*, 2009), danger signals produced directly by tissue damage (Srinivasan *et al.*, 2016) or mechanical force itself (Pereira *et al.*, 2011).

The VEGFR/Pvr and its ligand Pvf1 are also reasonable candidates for an early signal. Pvf1 becomes accessible to its receptor following wounding and epidermal Pvr is required for closure (Wu *et al.*, 2009). In the Pvr-deficient epidermis, a bulge of cytoplasm still accumulates at the wound edge (see Wu Fig. 2D), suggesting the wound edge cells can actually sense the presence of the wound. Similarly, in the JNK-deficient epidermis, leading edge cells protrude slightly into the wound gap although they are hindered by their continued synthesis of and attachment to the cuticle (see also Wu *et al.*, 2009, Fig. 2E). The experimental evidence for cell responsiveness (even if aberrant) in JNK-and Pvr-deficient larvae suggests that there are earlier signals produced and used to sense the presence of the wound. In embryos and pupae reactive oxygen species (ROS) are activated/produced very early after wounding to help recruit immune cells (Moreira *et al.*, 2010; Niethammer *et al.*, 2009; Razzell *et al.*, 2013). Although immune cells in larvae are not recruited through migration (Babcock *et al.*, 2008), it is possible that high levels of epidermal ROS serve as an early priming signal for other responses and pathways.

Physical force has long been proposed as a wound-induced signal (Enyedi and Niethammer, 2015; Harn *et al.*, 2017). Recently, techniques have been developed to evaluate membrane tension/ contraction using retraction velocity of membrane segments upon laser cutting (Colombelli and Solon, 2013). This was recently used to measure the contractile force of the actomyosin networks (Fernandez-Gonzalez and Zallen, 2013) and membrane tension during wound healing in the embryo (Kobb *et al.*, 2017). Although more technically challenging, it will be interesting to measure membrane tension before and after wounding both in control larvae and in larvae deficient for pathways known to be required for wound closure. Physical force/tension can also regulate the Hippo pathway (Aragona *et al.*, 2013; Dupont *et al.*, 2011; Rauskolb *et al.*, 2014). As with other pathways it will be interesting to test whether tension also regulates Hippo signaling during larval wound healing given that Yap/Yki rapidly translocates from the cytoplasm to the nucleus upon wounding (Tsai *et al.*, 2017). A tool that would be helpful is a way to deliver a defined local force to the larval cuticle so that both tissue damage and molecular pathway readouts can be systematically analyzed as a function of force and/or extent of damage. The mechanical filaments used in behavioral nociception studies (Kim *et al.*, 2012) may be adaptable for this purpose.

## Future directions - coordination of collective migration during wound healing

Larval epidermal wound healing is a form of collective migration because the epidermal cells involved migrate as a sheet, maintaining their connections to each other even as they heal the wound. Because of this, the responses of leading edge cells and follower cells need to be coordinated in order to close a wound. There is evidence already for distinct cellular responses of wound-edge and follower cells (Kwon *et al.*, 2010; Lesch *et al.*, 2010). Different wound closure genes can result in very distinct open wound phenotypes. Indeed, RNAi transgenes targeting Ced-12 (a PH-domain-containing adaptor protein) appear to have highly efficient follower cell migration and impaired leading edge migration (Lesch *et al.*, 2010). This suggests that the cellular responses of leader and follower cells, though potentially linked, can be genetically separated.

How are different signaling pathways activated in the leading cells or the followers? Wound-induced JNK signaling activation forms a gradient, with the cells closest to the wound showing the highest levels of JNK activation (Galko and Krasnow, 2004). One possibility is that different levels of JNK activation promote different cellular functions in a manner similar to the actions of developmental morphogens. In addition, during another Pvr-regulated collective migration process, border cell migration during oogenesis, the active (phosphorylated) Pvr was highly enriched at the front end of the leading cells compared to other regions of the border cell cluster (Janssens *et al.*, 2010). Higher Pvr activation in the leading cells increases faster endosome recycling, which maintains polarized distribution of Pvr activation (Wan *et al.*, 2013). It will be interesting to test if Pvr is activated preferentially in the leading cells or has other functions in the follower cells.

Another model is that different pathways are activated in different spatiotemporal domains and control responses appropriate to those times and locations. There is already some evidence for this-JAK/STAT signaling is activated farther away from the wound and later after wounding than JNK is (Lee *et al.*, 2017). This interplay regulates the spatiotemporal extent of wound-induced cell-cell fusion (Lee *et al.*, 2017). Ultimately, cells surrounding the wound need to integrate the combined inputs and crosstalk between the pathways activated at their location and over time. Exploring the crosstalk between signaling pathways (Fig. 2) and how they impact different cellular behaviors during wound healing will continue to be important moving forward.

## Future directions - genetic analysis of wound healing in real time

Larval wound healing serves as a powerful screening platform to identify genes that are required for wound healing (Baek *et al.*, 2010; Lesch *et al.*, 2010). With new advances in live imaging, it is now possible to monitor the whole healing processes of single-cell laser wounds (Kakanj *et al.*, 2016). Technical challenges still exist for visualizing larger wounds that take longer to heal. The development of live reporters for different signaling pathways (Bach *et al.*, 2007; Chatterjee and Bohmann, 2012; Kakanj *et al.*, 2016) open up the exciting possibility that it will soon be possible to monitor signaling outputs live in both space and time. However, it is still challenging to test at exactly when either before or after wounding these genes are important. Most of the loss-of-function studies performed to date turn off gene function throughout the entire larval stage. Therefore, it will be informative and important to develop tools that manipulate gene function at any desired time and location. For instance, advances in spatial (Luan *et al.*, 2006) and temporal (McGuire *et al.*, 2004; Osterwalder *et al.*, 2001) control of the Gal4/ UAS system may allow differential interrogation of the functions of genes in leading edge and follower cells. Another tool that will likely be useful moving forward is optogenetic manipulation of signaling events. For example, a recent study showed that Erk signaling can be regulated at specific times and regions during *Drosophila* embryogenesis (Johnson *et al.*, 2017). Similar approaches could be combined with the live imaging system (Kakanj *et al.*, 2016) to uncover the detailed signaling dynamics following wounding.

## Summary

Larval epidermal wound healing is a powerful platform to identify genes that are required for postembryonic wound healing. Many signaling pathways and their functions have been identified in this system. Although there are still many remaining questions to be explored, the knowledge gained from this system is likely to have implications in tissue repair and regeneration in other organisms since wound healing is highly conserved in metazoans.

## Figures and Tables

**TABLE 1:** WOUND CLOSURE GENES IN *DROSOPHILA* LARVAE

Class	Gene name	Human homolog	Functions	Reference
Actin regulators	*Rac1*	*RAC*	GTPase	(Baek *et al.*, 2010; Lesch *et al.*, 2010)
	*Cdc42*	*CDC42*	GTPase	(Lesch *et al.*, 2010)
	*Arp10*	*ACTR10*	Arp2/3 complex	(Lesch *et al.*, 2010)
	*Arp2*	*ACTR2*	Arp2/3 complex	(Lesch *et al.*, 2010)
	*SCAR*	*WAVE/WASF3*	Arp2/3 complex	(Lesch *et al.*, 2010)
	*zip*	*MYH10*	Nonmuscle myosin II heavy chain	(Kwon *et al.*, 2010)
	*Pak3*	*PAK3*	Target of Rac1	(Baek *et al.*, 2012)
	*chic*	*Profilin/PFN4*	Actin recycling	(Brock *et al.*, 2012)
	*Gγ1*	*GNG7*	Cell shape	(Lesch *et al.*, 2010)
	*Mbc*	*DOCK*	Phagocytosis	(Lesch *et al.*, 2010)
	*Ced-12*	*ELMO*	Phagocytosis	(Lesch *et al.*, 2010)

Signaling pathway	*Slpr*	*JNKKK/MAP3K11*	JNK signaling	(Lesch *et al.*, 2010)
	*Hep*	*JNKK2/MAP2K7*	JNK signaling	(Lesch *et al.*, 2010)
	*Bsk*	*JNK/MAPK8*	JNK signaling	(Galko and Krasnow, 2004; Lesch *et al.*, 2010)
	*Pvr*	*PDGFR/VEGFR*	Pvr signaling	(Wu *et al.*, 2009)
	*Pvf1*	*PDGF/VEGF*	Pvr signaling	(Wu *et al.*, 2009)
	*InR*	*Insulin receptor*	Insulin signaling	(Kakanj *et al.*, 2016)
	*Tor*	*MTOR*	TOR signaling	(Kakanj *et al.*, 2016)
	wts*	*LATS*	Hippo signaling	(Tsai *et al.*, 2017)
	ex*	*FRMD1*	Hippo signaling	(Tsai *et al.*, 2017)

Transcription factors	*DJun/Jra*	*JUN*	JNK signaling	(Lesch *et al.*, 2010)
	*DFos/kay*	*FOS*	JNK signaling	(Lesch *et al.*, 2010)
	foxo*	*FOXO*	Insulin signaling	(Kakanj *et al.*, 2016)
	*yki*	*YAP*	Hippo signaling	(Tsai *et al.*, 2017)
	*sd*	*TEAD*	Hippo signaling	(Tsai *et al.*, 2017)

Proteases	*Mmp1*	*MMP*	ECM Cleavage	(Stevens and Page-McCaw, 2012)
	*Mmp2*	*MMP*	ECM Cleavage	(Stevens and Page-McCaw, 2012)

* Note: Loss-of-functions of above genes show wound closure defects except for *foxo*, *wts* and *ex*. In contrast, gain-of-functions of *foxo, wts* and *ex* show wound closure defects, which are denoted by.

## Abbreviations used in this paper:

Arp2: actin-related protein 2
Arp10: actin-related protein 10
bsk: basket
Ced-12: cell death abnormality protein-12
chic: chickadee
DC: dorsal closure
D-Fos: *Drosophila* Fos
D-Jun: *Drosophila* Jun
ex: expanded
FA: focal adhesion
foxo: forkhead box sub-group O
Gγ1: G protein γ1
hep: hepmipterus
ILK: integrin-linked kinase
InR: insulin receptor
JAK: Janus kinase
mbc: myoblast city
Mmp: matrix metalloproteinase
Pak3: P21-activated kinase 3
PINCH: particularly interesting new cysteine-histidine-rich protein
Pvr: PDGF- and VEGF-receptor related
sd: scalloped
slpr: slipper
STAT: signal transducer and activator of transcription
Tor: target of rapamycin
VEGF: vascular endothelial growth factor
wts: warts
yki: yorkie
zip: zipper
